# Foodborne illness from tuba-tuba seeds among school-aged children, Philippines: a call for community education

**DOI:** 10.5365/wpsar.2025.16.1.1186

**Published:** 2025-03-20

**Authors:** Darren H Venturina, Apple Charm A Agulto, Alireza S Faiyaz, Ray Justin C Ventura, Mariz Zheila C Blanco-Payuyo, John Bobbie Roca

**Affiliations:** a Center for Health Development, Department of Health, Region IV-A, Calabarzon, Philippines.; b Region II Trauma and Medical Center, Department of Health, Cagayan Valley, Philippines.; c City Epidemiology, Health Statistics, and Disaster and Response Unit, Calabarzon, Philippines.; d Department of Health, Manila, Philippines.

## Abstract

**Objective:**

On 2 September 2023, the Regional Epidemiology and Surveillance Unit of the Department of Health’s Center for Health Development in Calabarzon, Philippines, received a report of foodborne illness due to the ingestion of tuba-tuba (*Jatropha curcas*) seeds in Talao Talao Village, Lucena City. The objective of this study was to describe the public health event.

**Methods:**

A descriptive study was conducted. Cases were defined as previously well individuals who developed at least one of the following symptoms after eating tuba-tuba seeds: vomiting, abdominal pain, diarrhoea, headache or dizziness. Health records were reviewed, and key informant interviews and environmental surveys were conducted.

**Results:**

Ten cases were identified, ranging in age from 10 to 12 years. The onset of symptoms ranged from 1 to 4 hours after consumption. Six of the cases were taken to the hospital, although two went home before being admitted; all recovered after 3 days. The most common symptom was vomiting (100%); other symptoms included abdominal pain, diarrhoea, dizziness and headache.

**Discussion:**

This investigation confirmed that tuba-tuba seeds were the cause of symptoms among school-aged children in Lucena City. To prevent similar events in the future, we recommend intensifying educational campaigns at both the community and school levels, as tuba-tuba is common in the area.

*Jatropha curcas*, commonly known as tuba-tuba in the Philippines, is an inedible perennial shrub that grows in tropical and subtropical regions. The name is derived from the Greek words “jatros” (doctor) and “trophe” (nutrition). *J. curcas* has a strong root system, and in the Philippines and elsewhere it is used in reforestation, soil rehabilitation projects and to reduce soil erosion. It is also common in and around towns, where it is widely used as a live fence, giving rise to the common name, *tubang bakod*. (*1*) Its seeds can be used as an insecticide, while its leaves are used in traditional medicine as a remedy for many ailments, including cough, fever, chills, headache, stomach ache, constipation, arthritis, fractures, muscle pain, dermatitis, haemorrhoids, infection with helminths and even to treat tumours. (*2*-*5*)

Although parts of the plant are known to have therapeutic properties, the seeds contain toxic compounds such as curcin (*2*) and curcanoleic acid, which when ingested can cause headache, dizziness and severe gastrointestinal symptoms, including vomiting, abdominal pain and diarrhoea. (*6*, *7*) Several episodes of foodborne illness have been attributed to the ingestion of tuba-tuba seeds in the Philippines and other countries where this plant is common. (*7*-*10*)

On 2 September 2023, the Regional Epidemiology and Surveillance Unit of the Center for Health Development, Department of Health, Calabarzon, received a report of foodborne illness due to the ingestion of tuba-tuba seeds in Talao Talao Village, Lucena City. Talao Talao is a coastal village in Quezon Province with a total population of 5234. (*11*) Lucena City, the capital city of Quezon Province, has a total population of around 278 924 as of the 2020 census. (*11*)

Given the widespread presence of *J. curcas* in the province and its potential health risks, an outbreak investigation was conducted by a team from the Department of Health’s Regional Epidemiology Surveillance Unit in Calabarzon. This report summarizes the outcome of that investigation and provides recommendations to prevent future occurrences.

## Methods

Following the report of a foodborne illness event in September 2023, disease surveillance officers and municipal personnel were deployed to Talao Talao on 8 September to carry out an initial investigation. A case was defined as a previously well individual who developed at least one of the following symptoms after eating tuba-tuba seeds in Talao Talao: vomiting, abdominal pain, diarrhoea, headache or dizziness. The investigation comprised a combination of medical records reviews, structured interviews and an environmental survey.

### Case investigation

On 8 September 2023, key personnel from a medical centre, local hospital and the Provincial Epidemiology and Surveillance Unit in Quezon were interviewed to gather historical data about similar events. During the investigation, pertinent data were also collected by interviewing cases and their legal guardians, and records were reviewed from the hospitals where cases were treated, with the help of the deployed disease surveillance officers. The data collected included sociodemographic characteristics (age, sex, place of residence, relationship to the index case, grade level), symptoms and clinical outcomes (if hospitalized or went home against medical advice, and whether recovered or not). Additionally, the Environmental Health and Sanitation Officer of the Lucena City Health Office and the village captain of Talao Talao were interviewed to determine whether they had relevant information about the public health event.

### Environmental assessment

An environmental survey of the site where the trees were located was conducted to determine the distribution and accessibility of the trees in the local area.

### Data analysis

This study employed a descriptive design. Case data were analysed using descriptive statistics; the environmental findings were summarized in a narrative review.

## Results

### Case identification and interviews

**Fig. 1:**
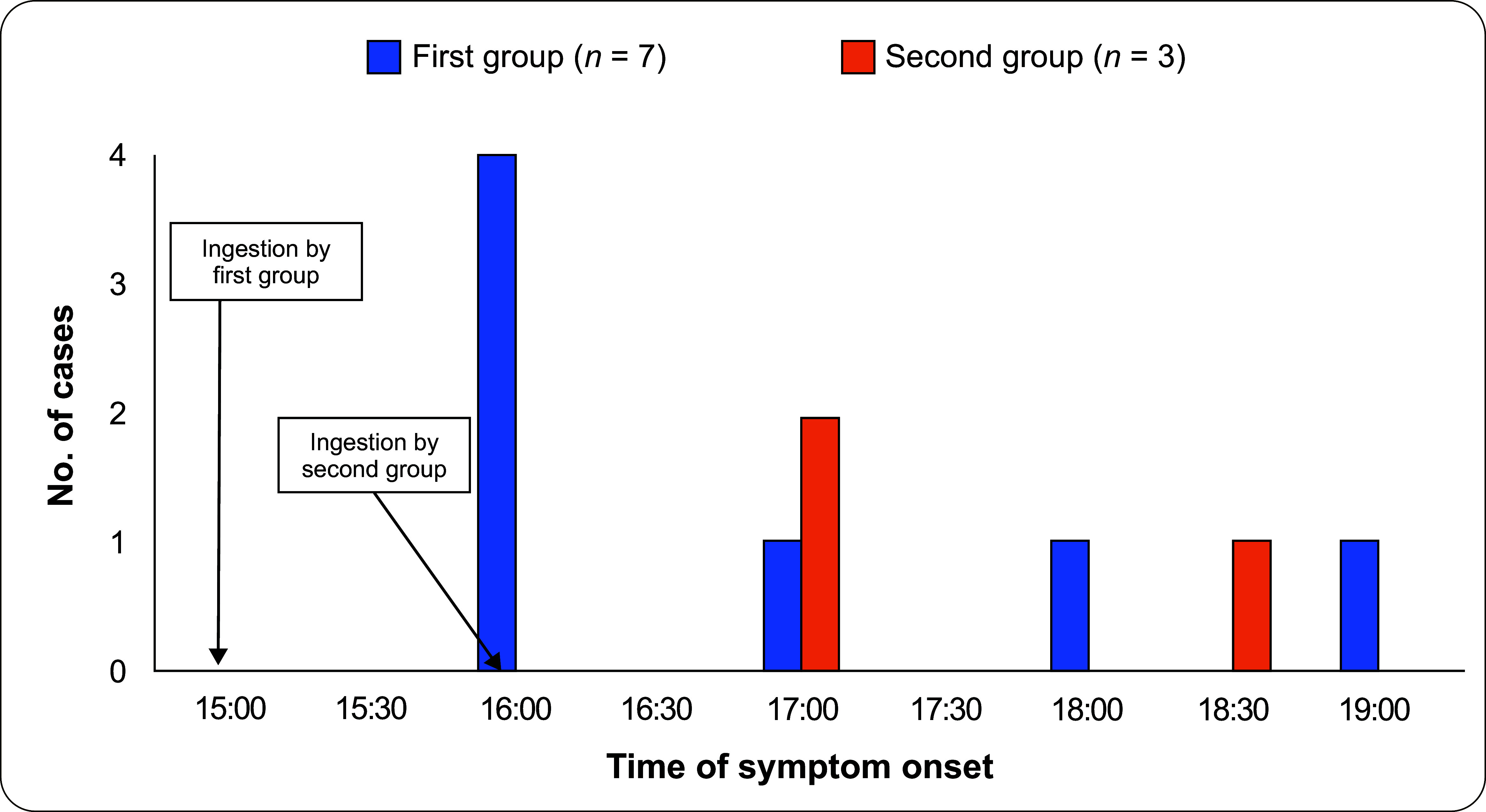
Foodborne illness from tuba-tuba (Jatropha curcas) seeds among 10 school-aged children, by time of symptom onset, Talao Talao Village, Lucena City, Philippines, 2 September 2023

Ten cases of foodborne illness due to ingestion of tuba-tuba seeds were identified, and a timeline of events was established (**Fig. 1**). On 2 September 2023, at around 13:00, a group of children aged 10–12 years went to Talao Talao Village to swim at a private resort. After a few hours and feeling hungry, seven children searched for trees bearing fruit. They found a tuba-tuba tree and, following a video they had seen on social media, harvested and consumed fresh tuba-tuba seeds at approximately 15:00, with some starting to show symptoms after 1 hour. They later shared the seeds with three additional friends who consumed them at around 16:00, and another friend started to show symptoms after 1 hour.

[**insert** Figure 1]

The onset of symptoms ranged from 1 to 4 hours after consumption, with an average onset of 1.75 hours. Six of the 10 children were taken to the hospital; four were admitted, but two went home against medical advice. All recovered after 3 days. All cases had vomiting. Additionally, some of the cases reported abdominal pain, diarrhoea, dizziness and headache (**Table 1**).

**Table 1:** Sociodemographic characteristics, clinical manifestations and outcome of foodborne illness due to ingestion of tuba-tuba (*Jatropha curcas*) seeds among 10 school-aged children, Talao Talao Village, Lucena City, Philippines, 2 September 2023

Characteristic	*n*(%)
**Age (years)**	
**10**	**1 (10)**
**11**	**5 (50)**
**12**	**4 (40)**
**Sought medical attention**	
**Yes**	**6 (60)**
**No**	**4 (40)**
**Admitted to hospital (among those who sought medical attention)**	
**Yes**	**4 (67)**
**No, went home against medical advice**	**2 (33)**
**No. of seeds ingested**	
**1–3**	**3 (30)**
**4–6**	**3 (30)**
**10–15**	**4 (40)**
**Incubation period (hours)**	
**1**	**6 (60)**
**2–4**	**4 (40)**
**Symptoms**	
**Vomiting**	**10 (100)**
**Abdominal pain**	**3 (30)**
**Diarrhoea**	**3 (30)**
**Dizziness**	**2 (20)**
**Headache**	**1 (10)**
**Hypovolaemic shock**	**1 (10)**
**Outcome**	
**Recovered**	**10 (100)**

In this event, it was noted that ingesting only one seed was needed to cause symptoms. Four children ingested at least 10 seeds, one of whom developed hypovolaemic shock. The four children who ingested fewer than six seeds were managed at home and did not experience serious complications.

### Interviews with health personnel

Interviews with personnel from the Provincial Epidemiology and Surveillance Unit revealed that foodborne illnesses from tuba-tuba seeds were recorded for the first time in Quezon Province during this outbreak investigation. Shortly after the Talao Talao incident, additional cases were reported in Wakas Village, Tayabas City, Quezon Province. In that instance, three cousins who were aged 4 years ingested the skin of the fruit, resulting in vomiting and diarrhoea. One child required medical evaluation at Quezon Medical Center.

Interviews with personnel from Quezon Medical Center, St. Anne General Hospital and the Lucena City Health Office indicated that the Talao Talao event was the first reported occurrence of foodborne illness in the municipality. The village captain of Talao Talao confirmed that tuba-tuba trees are common in the area, but this was the first documented event of foodborne illness associated with the trees.

### Environmental survey

The environmental survey revealed that the tuba-tuba trees were located on private property near the coast along Eco Road. The trees serve as a natural fence for the private resort. No warning signs regarding the dangers of consuming tuba-tuba seeds or fruit were present in the area. The trees were approximately 2 m tall, making the fruits easily reachable.

## Discussion

This outbreak of foodborne illness caused by the consumption of tuba-tuba seeds in Talao Talao was the first to be recorded in Lucena City; shortly afterwards, another unrelated outbreak occurred in nearby Tayabas City. In other countries, such as India, foodborne illness due to tuba-tuba ingestion is more commonly reported. (*6*-*10*) Most of the cases reported globally have occurred in children, and vomiting is the most common initial presenting symptom. (*6*, *7*, *12*) The predominance of gastrointestinal symptoms is likely attributed to the presence of curcanoleic acid, a potent gastrointestinal irritant and a constituent of tuba-tuba seeds. (*7*) Symptom onset is typically rapid; we observed a minimum of only 1 hour between ingestion and symptoms, although a previous study by Shah and Sanmukhani reported an incubation period as short as 15–20 minutes. (*7*) Other studies have established that ingestion of tuba-tuba seeds can lead to gastrointestinal symptoms lasting up to 72 hours or longer, (*13*) which can lead to hypovolaemic shock. In this outbreak, 1 of the 10 cases suffered hypovolaemic shock. The risks associated with ingesting tuba-tuba seeds should not be underestimated, especially in younger children who are more susceptible to dehydration.

Only one seed needed to be consumed to cause symptoms. Those who ingested fewer than six seeds were successfully managed at home and had no serious complications. Similar findings were noted in studies from India. (*6*, *7*, *12*) In this outbreak, children who ate six or more seeds were hospitalized, and among the four children who ingested at least 10 seeds, one had hypovolaemic shock. Ingestion of more than 10 seeds has been associated with more severe outcomes in a study from Israel, where two paediatric cases both developed hypovolaemic shock. (*13*) While it is tempting to conclude that the number of seeds ingested influences symptom severity, the weight of evidence does not yet support a dose–response relationship. For example, a large retrospective study from India (*6*) failed to find a dose–response relationship between the number of ingested seeds and the severity of symptoms. It is also noteworthy that in this event, the case who developed hypovolaemic shock was not the child who ingested the greatest number of seeds.

It is important to note that the children ingested tuba-tuba seeds after seeing a video clip on social media that led them to believe it was safe to do so. This finding should be a wake-up call to policy-makers to ensure that appropriate measures are in place to filter out dangerous content on social media platforms, especially content that is targeted at children during a period in their lives when they are explorative and impulsive. Other strategies that are important to consider include strengthening digital literacy programmes in schools and promoting awareness of the dangers of tuba-tuba through health clinics, and school-based and community health education programmes. A study on digital literacy showed that learning about the proper use of social media plays a role in preventing foodborne illness. (*14*) Signs warning of the dangers of consuming tuba-tuba seeds may also be effective. This recommendation is based on a study comparing the effectiveness of web and print media in communicating food safety practices to adolescents, which showed that the adolescents preferred the print-based media. (*15*)

This investigation has several limitations. First, we were not able to include laboratory testing to confirm the specific toxic compound responsible for the symptoms. However, the observed symptoms were consistent with those documented in the literature, supporting the conclusion that the seeds were the likely cause of the outbreak. In addition, given that the study relied on interviews with affected children and was conducted nearly a week after the incident, recall bias may have influenced the accuracy of reporting the number of seeds ingested. This potential inaccuracy likely impacted the study's ability to establish a clear dose–response relationship between seed consumption and symptom severity.

Despite these limitations, our investigation has confirmed that ingesting tuba-tuba seeds, regardless of the exact number, causes illness, reinforcing that these seeds should never be ingested. Although foodborne illness due to tuba-tuba seeds is not common in the area, the risks and toxic effects are sufficiently acute, especially in children, to warrant conducting intensified health and educational campaigns regarding the dangers of ingesting the seeds to prevent similar events in the future.
